# Using Osteopathic Manipulative Therapy to Treat Irritable Bowel Syndrome and Severe Refractory Gastroesophageal Reflux Disease: A Case Report

**DOI:** 10.7759/cureus.75811

**Published:** 2024-12-16

**Authors:** Timothy Johnson, Alek R Jahnke, Kelsey Reindel

**Affiliations:** 1 Department of Osteopathic Principles and Practice, Nova Southeastern University Dr. Kiran C. Patel College of Osteopathic Medicine, Tampa, USA; 2 Emergency Medicine, Hospital Corporation of America Florida Brandon Hospital, Brandon, USA

**Keywords:** alternative medical therapies, complementary medicine, gastroesophageal reflux disease (gerd), gastrointestinal disease, irritable bowel syndrome (ibs), musculoskeletal manipulation, osteopathic manipulative medicine (omm), osteopathic manipulative treatment (omt), osteopathic principles and practice (opp), visceral manipulation

## Abstract

Both Irritable Bowel Syndrome (IBS) and Gastroesophageal Reflux Disease (GERD) pose significant healthcare burdens on the general population of America. Though first-line medications are available, concomitant burdens of polypharmacy, side effects, and inadequate control exist. Osteopathic Manipulative Treatment (OMT) is a hands-on, physical manipulation technique that offers a personalized and direct approach to modifying the body’s neuromuscular and viscerosomatic activity leading to decreased symptomatic burden with minimal side effects. OMT comprises different modalities with significant versatility. They range from indirect, gentle techniques such as counterstrain to more direct, forceful techniques such as high-velocity low-amplitude (HVLA), each used as the situation demands.

This case report discusses a 28-year-old male patient who had struggled to control his ever-worsening GERD and IBS symptoms, despite consulting multiple physicians and undergoing treatment with various therapies. Even with adherence to the recommended medications and lifestyle changes, his symptoms had only been minimally reduced. This resulted in substantial distress, worsening his physical and emotional health.

A full-body OMT regimen was created and implemented to quell his symptoms at the source. This regimen was performed weekly for one month, with self-reported symptom prevalence evaluated from the first treatment until one month after the last treatment. Over the two months, the associated symptomatology became almost nonexistent. At the conclusion of the documented period, the individualized OMT regimen produced significantly greater symptomatic relief than any of the first-line medications used earlier.

## Introduction

America is plagued with various gastrointestinal complaints due to a culture with generally poor eating habits and a lack of exercise, in addition to genetic factors. This genetic predisposition can affect individuals' susceptibility to disease and their metabolism of medications. Amongst the most common diseases are irritable bowel syndrome (IBS), which affects ~15% of adults in North America, and Gastroesophageal Reflux Disease (GERD), which affects ~20% of adults in the United States (US) [1,2]. These diseases pose a significant symptomatic burden on the general population. Initial IBS therapy typically includes lifestyle and dietary modification. However, if this does not effectively control the disease, further treatment is based on the type of IBS. IBS Constipation type is managed with soluble fiber, such as psyllium, followed by polyethylene glycol, lubiprostone, or linaclotide as needed. Conversely, IBS Diarrheal type is managed with anti-diarrheals such as loperamide, followed by bile acid sequestrants such as cholestyramine [3]. Similarly, GERD treatment often involves dietary modification along with various antacid medications, of which proton pump inhibitors (PPIs) are the most common [2]. 

Unfortunately, due to a lack of accessible healthcare, finances, side effects of medicines, comorbidities, and genetic variations, these treatments only work for some patients. Around 40% of patients with GERD fail to achieve complete symptomatic relief while using PPIs, the gold standard of treatment [4]. As the disease progresses, the risk of developing gastric and likely laryngeal, pharyngeal, oral and lung cancers also increases [5]. However, even when attempting to subdue this disease, treatment with PPIs often presents with a variety of undesired side effects. These include increased incidence of enteric infections, microscopic colitis, inflammatory bowel disease, acute interstitial nephritis, and significant malabsorption (often magnesium, calcium, vitamin B12, and iron) [6]. The need for an alternative treatment is apparent. 

Osteopathic Manipulative Treatment (OMT) has been shown to effectively reduce symptomatic GERD through a variety of modalities in numerous patients with minimal side effects. By balancing the parasympathetic and sympathetic stimulation and freeing the patient’s diaphragm to improve lymphatic-vascular flow, OMT can quell the GERD-related issues on multiple fronts [7,8]. One case report showed that individualized OMT was able to treat severe GERD refractory to PPIs [9]. 

The pathophysiology of autonomic dysregulation and lymphatic-vascular restriction that precipitate GERD symptoms are also involved in provoking IBS. Although the data is limited and of a smaller sample size, preliminary studies with OMT in IBS patients have shown similar positive benefits in terms of symptomatic relief [10].

OMT has a role in reducing the patients’ burdens of polypharmacy, side effects and failed treatments. Along with very few contraindications and low cost, this treatment can help people of all socioeconomic strata and from many different paths of life. 

## Case presentation

A 28-year-old Caucasian male patient presented with a history of IBS and GERD refractory to PPI treatment leading to significant exercise intolerance and emotional distress. He reported that this problem persisted for years and was gradually worsening. He stated that it worsened with cardiovascular activity and led to nausea, vomiting, diarrhea, palpitations, lightheadedness, hyperhidrosis, and hot flashes. The episodes of vomiting provided temporary relief to his symptoms but they recurred shortly thereafter.

These symptoms had significant lifestyle implications and limited the many sports he had enjoyed for years. He had undergone a thorough evaluation by a cardiologist and a gastroenterologist, in addition to his primary care physician. The cardiac evaluation revealed a normal physical exam, a benign cardiac history, and a normal EKG without any evidence of arrhythmia, ischemia, or an infarct. The cardiologist determined that his symptoms were not of cardiac origin and recommended a follow-up with gastroenterology. The patient's work-up with a gastroenterologist over four visits consisted of multiple normal physical exams, in addition to a normal CBC and basic metabolic panel (BMP). He also underwent an endoscopy and colonoscopy which were normal, without any evidence of bleeding, ulcers, Barrett's, gastritis, esophagitis, polyps, masses, or colitis. He was eventually diagnosed with GERD and IBS. His gastroenterologist prescribed extended trials of various PPIs including pantoprazole and omeprazole. Both trials only produced a slight improvement in symptoms at rest. While undergoing OMT, the patient continued his omeprazole as prescribed. He had been taking it regularly for the previous three months. He denied taking any other medications or using any supplements. During the first month of treatment, there was no change in his exercise regimen. The patient continued his weightlifting exercises two to three times per week and avoided intense cardiovascular exercise.** **

The patient’s past medical history included the diagnosis of GERD at the age of 10, occasionally leading to emesis after strenuous exercise or sports. As a child, this was managed successfully by limiting fatty foods in the diet and avoiding strenuous exercise around mealtimes. However, adult-onset exercise intolerance presented itself at the age of 24. Aside from this, the patient had no concerning medical history or allergies.

We hypothesized that symptomatic relief in GERD and IBS can be achieved with a personalized OMT approach to suppress the overactive parasympathetic and sympathetic gastrointestinal innervation, along with lymphatic congestion.

The treatment and follow-up included numerous direct OMT modalities, in several organ systems, all designed to achieve the same goal synergistically (Table 1).

**Table 1:** The OMT procedure summary repeated weekly in the treatment of IBS and GERD OMT: Osteopathic manipulative treatment; IBS: Irritable bowel syndrome; GERD: Gastroesophageal reflux disease; T: Thoracic; L: Lumbar

Treatment/Modality	Location
Soft tissue	Cervical, thoracic, lumbar paraspinal musculature
High Velocity Low Amplitude	Cervical, thoracic, lumbar vertebrae
Decompression (suboccipital release)	Occipito-atlantal
Sympathetic nerve inhibition	T5-L2
Parasympathetic nerve inhibition	Sacrum
Direct myofascial release	Celiac, superior mesenteric and inferior mesenteric ganglia
Direct myofascial release	Thoracic inlet
Lymphatics	Doming of the diaphragm

As the patient was young and otherwise healthy, multiple direct techniques were implemented. This treatment was repeated every week for a month (5/9/23, 5/16/23, 5/23/23, 5/30/23). Each treatment session lasted between 30 and 60 minutes and was completed by Dr. Johnson (student) with physician supervision. Although the writing of this report involved authors from various institutions, the trial was completed while all the authors were collaborating at the same location as medical students or professors of Nova Southeastern University. 

The results included reduction in symptoms that appeared after the first treatment with the patient reporting a significant decrease in nausea, lightheadedness, and overall exercise intolerance for two consecutive workouts during the week. However, the symptoms returned the following week. The patient again reported significant improvement in the same symptoms for four to seven days following the second treatment. After a month, when all four treatments were completed, the patient continued to note symptomatic improvement with a significant reduction in the number of episodes of nausea, emesis, and lightheadedness (Table 2). This led to an overall increase in the patient's ability to tolerate exercise with improved physical and emotional health. These results lasted for roughly two weeks before notable symptomatology began to reappear.

**Table 2:** Self-reported average instances of symptomatic episodes per week experienced before, during, and after the implementation of OMT OMT:  Osteopathic manipulative treatment

Timeline	Nausea	Vomiting	Diarrhea	Lightheadedness	Hyperhidrosis	Hot flashes
Before treatment	3	3	4	3	4	4
Week after treatment #1	3	3	3	3	4	4
Week after treatment #2	2	2	3	2	2	2
Week after treatment #3	2	1	2	2	2	2
Week after treatment #4 (final treatment)	1	0	1	2	1	1
Two weeks after final treatment	1	0	1	1	0	0
One month after final treatment	2	0	2	1	0	0

After the one month of follow up and symptomatic documentation ended, the patient sought treatment sporadically, as needed (three times over the next two months). With continued treatment, the duration of symptom remission increased. Additionally, the patient was questioned at each appointment and there were no reported side effects over the entire duration of treatment

## Discussion

The US is a country with a significant burden of obesity, poor dietary choices, and lackluster lifestyle modifications as a population, causing an epidemic of GERD and IBS. Regarding treatment in the US, 25% of the graduating physicians are Doctors of Osteopathic Medicine, and this figure is rising [11]. In addition, any MD with OMT training can also practice under his/her own license. This vast pool of underutilized resources can make a major difference. 

What may seem like common diseases from the perspective of general medicine can have far more deleterious effects when evaluating the patient holistically. This patient had been battling recurrent episodes of gastrointestinal (GI) irritation since childhood. However, despite his self-stated adequate workup through the primary physician, two specialists, and lifestyle modifications, he continued to struggle with his disease. Although he was free from the burden of side effects due to PPIs or the worry of polypharmacy that many endure, he struggled to be as physically active as most 28-year-olds are. Unlike many, he wanted to make changes to help himself. However, he needed help from alternative therapies to reach that goal. 

With the knowledge of the preliminary successes in treating GERD and IBS with OMT, along with a basic understanding of the pathophysiology of his diagnoses, we were able to create a personalized treatment regimen that blocked his problem at the source [7-10]. This was done by treating three separate but intertwined physiological systems, hypothesized to be aggravating his symptoms - sympathetic and parasympathetic imbalance along with lymphatic congestion. Since GERD and IBS have upper, middle, and lower GI effects, this treatment aimed to balance autonomic innervation and free lymphatic flow to the entire GI tract.

The overactive parasympathetics were normalized via occipito-atlantal decompression and sacral inhibition [12]. Similarly, viscerosomatic normalization was achieved through thoracic and lumbar soft tissue followed by HVLA. Additional sympathetic de-escalation was achieved through direct myofascial release of the celiac, superior, and inferior mesenteric ganglia, along with sympathetic inhibition of T5-L2. Finally, the lymphatic flow was stimulated by opening the thoracic inlet, followed by doming of the respiratory diaphragm [13]. 

This focused manipulation and rebalancing of the patient’s autonomic nervous and lymphatic systems granted him symptomatic relief without any adverse effects. Although the effects were not long standing at first, they endured as the follow-ups continued. This exploratory treatment facilitated better symptomatic relief and quality of life than extended trials of two different first line medications (omeprazole and pantoprazole). 

This case report demonstrated a preliminary successful, alternative treatment to symptomatic GERD and IBS. Although this trial involved only one individual over a brief period of time, its marked success indicates that more evaluation is needed. If replicated, this regimen has the potential to provide improved symptomatic relief to a disease hindering the quality of life of thousands of Americans each year [1,2]. With the variance across patients, a tailor-made, personalized approach will definitely produce optimal results. If ~40% of patients with GERD fail to achieve complete symptomatic relief with PPIs, there needs to be a larger emphasis on newer treatment options [4]. With its minimal side effects and affordable cost along with an ever-growing population of providers, OMT could have a significant positive impact on the future healthcare of the US population. 

## Conclusions

By applying widely accepted principles of the autonomic nervous system and understanding the pathophysiology of GERD and IBS, this preliminary report shows that OMT has substantial benefits in offering patients symptomatic relief. In general, OMT has an exceptionally low side effect profile and minimal cost burden, and with a growing number of providers, it can translate into a cheap, safe, and widely accessible treatment for those suffering from these disorders. More case studies and clinical trials are needed to further establish the benefits of OMT on GERD and IBS in a larger patient population, along with comparing the efficacy of specific OMT modalities on different target body regions, disease comorbidities, and severities.
